# Insights on Immune Function in Free-Ranging Green Sea Turtles (*Chelonia mydas*) with and without Fibropapillomatosis

**DOI:** 10.3390/ani11030861

**Published:** 2021-03-18

**Authors:** Justin R. Perrault, Milton Levin, Cody R. Mott, Caitlin M. Bovery, Michael J. Bresette, Ryan M. Chabot, Christopher R. Gregory, Jeffrey R. Guertin, Sarah E. Hirsch, Branson W. Ritchie, Steven T. Weege, Ryan C. Welsh, Blair E. Witherington, Annie Page-Karjian

**Affiliations:** 1 Loggerhead Marinelife Center, Juno Beach, FL 33408, USA; shirsch@marinelife.org; 2 Department of Pathobiology and Veterinary Science, University of Connecticut, Storrs, CT 06269, USA; milton.levin@uconn.edu; 3 Inwater Research Group, Jensen Beach, FL 34957, USA; cmott@inwater.org (C.R.M.); mbresette@inwater.org (M.J.B.); rchabot@inwater.org (R.M.C.); jguertin@inwater.org (J.R.G.); sweege@inwater.org (S.T.W.); rwelsh@inwater.org (R.C.W.); bwitherington@inwater.org (B.E.W.); 4 Gumbo Limbo Nature Center, Boca Raton, FL 33432, USA; cbovery@ci.boca-raton.fl.us; 5 Infectious Diseases Laboratory, University of Georgia, Athens, GA 30602, USA; crg@uga.edu (C.R.G.); britchie@uga.edu (B.W.R.); 6 Harbor Branch Oceanographic Institute, Florida Atlantic University, Fort Pierce, FL 34946, USA; cpagekarjian@fau.edu

**Keywords:** ChHV5, ChHV6, ELISA, herpesvirus, lung-eye-trachea disease, lymphocyte proliferation, marine turtle, mitogen, natural killer cell, seroprevalence

## Abstract

**Simple Summary:**

Sea turtles are susceptible to several herpesviruses that are linked to dermatologic diseases, including fibropapillomatosis (FP) and lung-eye-trachea disease. Aside from obvious skin lesions, a number of other sublethal impacts occur in response to these diseases, such as reduced immune function. In this study, we found no relationship between disease presence or severity and T-cell proliferation in green turtles from Florida, USA, at least until the moderate stages of FP; however, natural killer cell activity, a measure of innate immune function, was significantly reduced in turtles with FP compared to tumor-free individuals. This is the first study to examine natural killer cell activity in relation to FP, improving upon our understanding of altered immune system function associated with this disease.

**Abstract:**

Chelonid alphaherpesviruses 5 and 6 (ChHV5 and ChHV6) are viruses that affect wild sea turtle populations. ChHV5 is associated with the neoplastic disease fibropapillomatosis (FP), which affects green turtles (*Chelonia mydas*) in panzootic proportions. ChHV6 infection is associated with lung-eye-trachea disease (LETD), which has only been observed in maricultured sea turtles, although antibodies to ChHV6 have been detected in free-ranging turtles. To better understand herpesvirus prevalence and host immunity in various green turtle foraging aggregations in Florida, USA, our objectives were to compare measures of innate and adaptive immune function in relation to (1) FP tumor presence and severity, and (2) ChHV5 and ChHV6 infection status. Free-ranging, juvenile green turtles (*N* = 45) were captured and examined for external FP tumors in Florida’s Big Bend, Indian River Lagoon, and Lake Worth Lagoon. Blood samples were collected upon capture and analyzed for ChHV5 and ChHV6 DNA, antibodies to ChHV5 and ChHV6, in vitro lymphocyte proliferation using a T-cell mitogen (concanavalin A), and natural killer cell activity. Despite an overall high FP prevalence (56%), ChHV5 DNA was only observed in one individual, whereas 20% of turtles tested positive for antibodies to ChHV5. ChHV6 DNA was not observed in any animals and only one turtle tested positive for ChHV6 antibodies. T-cell proliferation was not significantly related to FP presence, tumor burden, or ChHV5 seroprevalence; however, lymphocyte proliferation in response to concanavalin A was decreased in turtles with severe FP (*N* = 3). Lastly, green turtles with FP (*N* = 9) had significantly lower natural killer cell activity compared to FP-free turtles (*N* = 5). These results increase our understanding of immune system effects related to FP and provide evidence that immunosuppression occurs after the onset of FP disease.

## 1. Introduction

Green sea turtles (*Chelonia mydas*) are considered indicators of marine ecosystem health [1,2,3,4]. They play important roles in seagrass bed resiliency by preventing overgrowth and reducing nutrient input into these areas [5]. Green turtle populations in the northwestern Atlantic Ocean suffered substantial population declines prior to the 20th century due to overexploitation of eggs and adults for human consumption, and are subject to modern threats including entanglement in fishing gear, habitat loss, beach-front lighting, boat strikes, and pollution [3,6,7,8]. Despite their current threatened status, green turtle populations in Florida, USA appear to be recovering due to conservation efforts, evidenced by increasing nest counts, hatchling production, and captures of immature individuals in foraging areas [3,7,9,10,11,12]. Although these population trends are encouraging, immature green turtles in Florida have a high prevalence (>50%) of fibropapillomatosis (FP) [13,14,15,16,17,18], a transmissible tumor disease associated with chelonid alphaherpesvirus 5 (ChHV5) infection [19]. Extensive research has been conducted in an attempt to better understand FP, yet etiology, pathogenesis, and transmission are still not well understood. Turtles inhabiting nearshore, shallow-water embayments tend to express the highest FP prevalence. Environmental cofactors including agricultural runoff, exposure to biotoxins and toxicants, habitat degradation, warm water temperatures, and UV radiation have been suggested to play a role in FP pathogenesis and maintenance within green turtle populations [17,19,20,21,22,23,24,25,26,27]. To date, however, no studies have identified confirmed causal relationships between environmental cofactors and FP occurrence.

Since its discovery in the Florida Keys in 1937 [28] and in Hawaii in 1958 [29], FP has become a panzootic in green turtles with observations in all seven sea turtle species in numerous populations around the globe [30,31,32,33,34,35,36,37]. ChHV5 is likely horizontally transmitted via shedding of infected epithelial cells, and possibly in urine and/or bodily secretions [21,38,39,40,41,42]. When FP tumors are present, depending on tumor size, extent, and anatomic location, they can cause physical alterations that lead to reductions in afflicted turtles’ ability to see, breathe, swim, dive, forage, and avoid predators [43]. ChHV5 nucleic acids can be detected in biological samples using polymerase chain reaction (PCR). Previous exposure to, and presumptive infection with ChHV5 can be identified via detection of circulating antibodies in sea turtle blood samples using enzyme-linked immunosorbent assays (ELISA) [4,44]. Changes in blood-based indicators of health have been observed in turtles with FP, including anemia, hypoproteinemia, leukopenia/immunosuppression, and other biochemical alterations [15,17,29,45,46,47,48,49,50,51]. More specific associations of FP with afflicted turtles’ immune function include decreased T- and B-cell function, suggesting impacts to cell-mediated and humoral immunity (i.e., adaptive immunity). It remains unknown, however, whether these immunomodulatory alterations are a cause of or sequela to the onset of FP disease [27,36,46,47].

Another disease-causing herpesvirus that affects sea turtles is chelonid alphaherpesvirus 6 (ChHV6) [52], formerly known as lung-eye-trachea virus (LETV), which was first recognized in 1975 in 15–20-month-old, maricultured green turtles residing at the Cayman Turtle Farm (Grand Cayman, British West Indies). Clinical signs included respiratory and buoyancy abnormalities with caseous exudate surrounding the eyes, glottis, and trachea, and a mortality rate of up to 38% [53]. Since its discovery, lesions associated with ChHV6 infection have not been reported in free-ranging turtles; however, antibodies to ChHV6 have been detected in juvenile green turtles from Florida with seroprevalence rates of 10–22%, depending on location [54,55]. Nesting loggerheads (*Caretta caretta*; 75% seroprevalence: 3/4 from Melbourne Beach, Florida) and green turtles (100% seroprevalence: 9/9 from Melbourne Beach, Florida; 15% seroprevalence: 6/41 from Juno Beach, Florida) have also tested positive for ChHV6 antibodies via ELISA [4,55], but negative for circulating ChHV6 DNA via PCR [4]. Despite exposure to ChHV6, abnormalities in blood health analytes have not been observed in turtles with ChHV6-specific antibodies [4]. To build upon previous studies associating sea turtle host immunity to gross and molecular diagnostic measures of ChHV5 and ChHV6 infection, the objectives of this study were to compare innate and adaptive immune function in relation to (1) FP tumor presence and severity, and (2) ChHV5 and ChHV6 infection status in free-ranging, juvenile green turtles from three foraging aggregations across Florida.

## 2. Materials and Methods

### 2.1. Sampling Sites

#### 2.1.1. Big Bend Region, Florida

**Figure 1:**
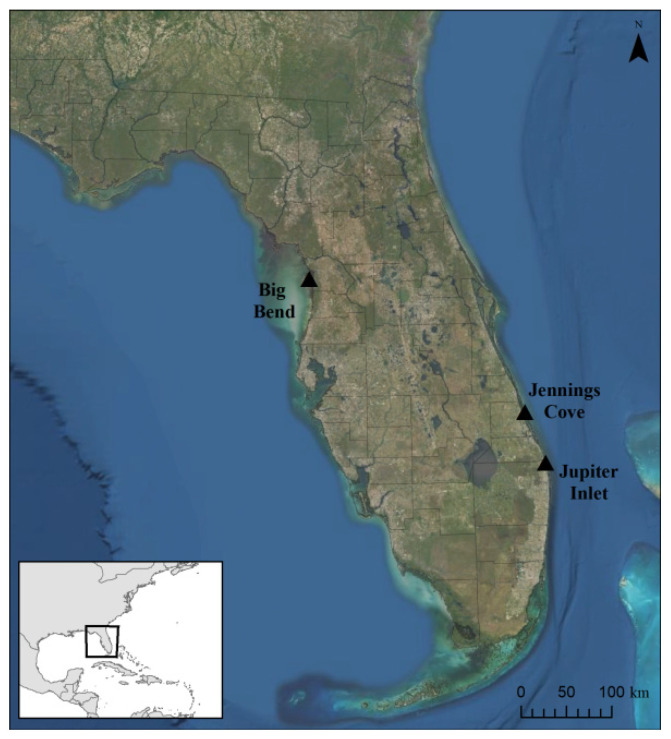
Locations of green turtle (*Chelonia mydas*) sampling sites across Florida, USA: Big Bend Region, Jennings Cove in the Indian River Lagoon, and the Jupiter Inlet in the northern portion of the Lake Worth Lagoon.

The coastal waters of Florida’s Big Bend region (Figure 1; hereafter referred to as Big Bend) extend from the eastern boundary of the Apalachicola–Chattahoochee–Flint River Watershed to Anclote Key in Pinellas County. This area serves as an important foraging habitat for juvenile green and Kemp’s ridley (*Lepidochelys kempii*) sea turtles as it contains the second largest expanse of seagrass habitat in the eastern Gulf of Mexico [18,56,57], with primarily natural land coverage, conservation protection of much of the coastal and inland areas, low human population density, and minimal anthropogenic threats (e.g., boating, eutrophication, and pollution) [58,59]. Despite this seemingly pristine environment, loss of oyster beds and seagrasses and a high FP prevalence rate (60–70%) in green turtles occur in this area [18,57,59].

#### 2.1.2. Jennings Cove, Indian River Lagoon, Florida

The Indian River Lagoon (IRL) spans 250 km of Florida’s east coast, was described as one of the most biodiverse estuaries in North America, and has been designated as an Estuary of National Significance [60,61]. This system has been impacted by human population growth, species loss, nutrient runoff, pollutants, and harmful algal blooms. Poor water quality and declines in ecological and biological integrity have occurred in the area due primarily to nutrient-rich runoff and habitat loss [62,63,64]. More than 50 point-source waste sites are permitted to discharge pollutants into the IRL including emissions from power plants, brine discharges, septic tanks, and water treatment plants [61]. The prevalence of FP in green turtles captured at Jennings Cove in the southern portion of the IRL (Figure 1) has varied with no detectable trend over the last 10–15 years, with annual prevalence rates ranging from 33 to 90% (mean ± standard error of 68 ± 6%) [65].

#### 2.1.3. Jupiter Inlet, Lake Worth Lagoon, Florida

The Jupiter Inlet (Figure 1) serves as the northernmost boundary of the Lake Worth Lagoon, which is a heavily urbanized waterway spanning ~32 km in Palm Beach County, Florida. Research on the Jupiter Inlet is lacking; however, the primary environmental issues in the Lake Worth Lagoon system are related to untreated stormwater runoff and septic tank overflow, which can drain into the lagoon, subsequently leading to reductions in water clarity, salinity fluctuations, and excess nutrient deposition [66,67]. Over the last 10–15 years, underground storm water treatment areas have been installed to filter water before it reaches Lake Worth Lagoon. This is part of an overall restoration plan that includes mitigation measures to enhance seagrass, oyster beds, and mangrove habitats in the lagoon. Juvenile green turtles are abundant in the more southern areas of Lake Worth Lagoon and exhibit a high prevalence (~50%) and severity of FP [16], but little is known about FP rates in turtles from the more northern areas of the lagoon (i.e., Jupiter Inlet).

### 2.2. Animal Capture and Sample Collection and Processing

Green turtles were captured using dip nets, tangle nets, and/or the rodeo method, depending on habitat and environmental conditions [68]. Turtles from Big Bend, Jennings Cove, and Jupiter Inlet were sampled from 27 to 30 August 2020, 20 September 2018 to 8 October 2020, and 17 to 18 September 2020, respectively. Captured turtles were brought onboard the vessel for data collection. Prior to sample collection, turtles were externally examined [69,70], weighed and measured (standard straight carapace length, SCL), assessed for subjective body condition (i.e., emaciated, thin, good, and robust), and Fulton’s body condition index (BCI) was calculated [6]. A complete external visual exam was conducted on all turtles. This included observations for the presence or absence of leeches (*Ozobranchus* spp.), leech cocoons, barnacles, flipper and carapace damage, and visual assessment and enumeration of FP tumors (i.e., total tumor number). Flipper and carapace damage were defined as missing >5% of the flipper or carapace and/or an injury caused by predation, anthropogenic impact, or abnormal development. Tumors were measured and recorded on a standardized tumor score sheet. Each turtle was assigned a tumor severity category based on tumor burden (0: no tumors; 1: mildly afflicted; 2: moderately afflicted; 3: severely afflicted) [29]. Blood (~10–20 mL; <1% of body weight) was sampled from the external jugular vein using sodium heparin and serum separator Vacutainer^®^ (Becton, Dickson, and Co., Franklin Lakes, NJ, USA) blood collection systems fitted with 21–25-gauge, 1 inch needles, as appropriate based on turtle size. The venipuncture site was swabbed with alternating applications of povidone iodine and 70% isopropyl alcohol prior to and after blood collection. Blood samples were immediately chilled on ice in the field until return to the laboratory (<8 h). An aliquot (~100 µL) of well-mixed whole blood was placed into a separate cryovial and stored in an ultralow freezer (−80 °C) for up to six months prior to DNA extraction for PCR analysis. The remaining whole blood in the sodium heparin vacutainers was shipped overnight on ice packs to the University of Connecticut. Serum from the serum separator tubes was harvested after centrifugation at 1318× *g* (3400 rpm) for 10 min and stored in an ultralow freezer for up to 6 months prior to serological analysis.

All turtles were tagged with metal flipper (Inconel^®^) and passive integrated transponder (PIT) tags for identification. Tagging sites were swabbed with povidone iodine and 70% isopropyl alcohol prior to and after tag application. After completion of sampling and tagging, turtles were released back into the water at or near the site of capture.

### 2.3. Molecular Diagnostics for ChHV5 and ChHV6 DNA

Quantitative PCR (qPCR) for ChHV5 DNA was performed at Florida Atlantic University’s Harbor Branch Oceanographic Institute in Fort Pierce, Florida. First, genomic DNA (gDNA) was extracted from thawed whole blood samples using the DNeasy Blood and Tissue kit according to the manufacturer’s instructions (Qiagen, Hilden, Germany). Concentrations of extracted gDNA samples were quantified using a NanoDrop 2000c (Thermo Fisher Scientific, Waltham, MA, USA) (units: μg/μL) spectrophotometer, and the ratio of absorbance at 260 and 280 nm was used to assess gDNA purity. Extracted gDNA samples were assessed for the presence of a ChHV5 UL30 gene segment using a singleplex, hydrolysis probe-based qPCR and the methodologies described in detail by Page-Karjian et al. [40]. Quantitative PCR reactions were conducted using an AriaMx Real-Time PCR System (Agilent, Santa Clara, CA, USA), and qPCR data were analyzed with AriaMx software (Agilent, Version 1.3). All ChHV5-positive qPCR products were purified using the QIAquick PCR Purification Kit (Qiagen) and subjected to Sanger sequencing (Genewiz) using 5 μM of ChHV5 UL30 forward primer. Sequences obtained were compared to those deposited in the National Center for Biotechnology Information GenBank database using BLAST software [71]. Aligned sequences with ≥97% identity to the sample sequence were considered a match.

Aliquots of frozen whole blood were shipped overnight on dry ice to University of Georgia’s Infectious Diseases Laboratory (UGA IDL; Athens, GA, USA). Genomic DNA was extracted from these samples using the methodology outlined above. Extracted gDNA samples were analyzed using a singleplex, hydrolysis probe-based qPCR assay that targets a unique 112 bp ChHV6 DNA amplicon, using standard operating procedures and controls for the assay. Detailed methodology is provided in Page-Karjian et al. [4].

### 2.4. ELISA Detection of Antibodies to ChHV5 and ChHV6 Peptides

Separated serum samples were analyzed for antibodies to ChHV5 and ChHV6 peptides at the UGA IDL. To evaluate for infection by ChHV5 and ChHV6 in a turtle’s immunologically detectable past, samples were analyzed in triplicate using ELISAs that test for antibodies to ChHV5 and ChHV6 purified synthetic peptide antigens. Both ELISA assays were developed and validated based on modifications of previously published protocols for ChHV5 and ChHV6 assays [54,72] and were performed using the laboratory’s standard operating procedures with negative and positive control sera. Detailed methodology is provided in Page-Karjian et al. [4].

### 2.5. Isolation of Peripheral Blood Mononuclear Cells

At the University of Connecticut (Storrs, CT, USA), whole blood from the sodium heparin tubes was mixed 1:1 with an equal volume of Hank’s Balanced Salt Solution (HBSS; Thermo Fisher Scientific). Peripheral blood mononuclear cells (PBMCs) were isolated by density gradient centrifugation using Ficoll-Paque plus (Amersham Biosciences, Uppsala, Sweden; 1.077 g/mL) for 30 min at 600× *g*. The interface containing the PBMCs was re-suspended in complete RPMI and washed twice in complete RPMI. PBMCs were enumerated and their viability was assessed using the exclusion dye trypan blue and light microscopy. Complete RPMI consisted of RPMI 1640 (with 2 mM L-glutamine; Thermo Fisher Scientific) supplemented with 1 mM sodium pyruvate, 100 mM nonessential amino acids, 10 mM HEPES, 50 U/mL penicillin, 50 µg/mL streptomycin, 0.25 µg/mL fungizone, and 10% fetal bovine serum (all from Thermo Fisher Scientific).

### 2.6. Lymphocyte Proliferation

Mitogen-induced lymphocyte proliferation was evaluated in vitro as previously described [73], with some modifications. Briefly, PBMCs in complete RPMI were plated (2 × 10^5^ cells/well) in a 96-well flat-bottom plate (Falcon; Becton, Dickinson, and Co.) in triplicate for each mitogen concentration. Cells were maintained for 96 h in an incubator at 28 °C and humidified atmosphere with 5% CO_2_. The T-cell mitogen concanavalin A (ConA, C5275, Millipore Sigma, St. Louis, MO, USA) at 1 (suboptimal) and 10 μg/mL (optimal) was used. Lymphocyte proliferation was evaluated as the incorporation of 5-bromo-20-deoxyuridine (BrdU), a thymidine analogue, added for the last 18 h of incubation, and further detected with a monoclonal antibody and a colorimetric enzymatic reaction (Cell Proliferation colorimetric ELISA BrdU, RocheDiagnostics GmbH, Mannheim, Germany) as per manufacturer’s instructions using an ELISA plate reader (Multiskan EX v.1.0) at 450 nm with a reference wavelength of 690 nm. Data are reported as:

(1) $$\mathrm{Stimulation}\;\mathrm{index}\;=\;\left(\frac{Mitogen\;optical\;density}{Unstimulated\;optical\;density}\right)\;\times \;100\;$$

Results with cell viability percentages <80% were excluded from statistical analyses.

### 2.7. Natural Killer Cell Activity

For green turtles captured in Big Bend, natural killer cell activity was evaluated in vitro as previously described [73,74] with some modifications. Briefly, 1 mL of YAC-1 (TIB-160™, ATCC, Manassas, VA, USA) target cells was incubated with 10 µL of 3 mM 3,3′-dioctadecyloxabocyanine perchlorate (DiO, Molecular Probes, Grand Island, NY, USA) dissolved in DMSO, and incubated for 20 min at 37 °C in 5% CO_2_, followed by two washes in complete RPMI. The target cells were then re-suspended in complete RPMI. PBMCs (effector cells) were adjusted to 1 × 10^6^ cells/mL and target cells were added to achieve effector:target (E:T) ratio of 50:1 [74]. The E:T mixtures were centrifuged for 30 s at 220× *g* and further incubated for 150 min at 28 °C in 5% CO_2_. All tests were performed in duplicate.

Following centrifugation at 220× *g* for 10 min at 4 °C, the supernatant was discarded and the cells were re-suspended in 200 µL of phosphate buffered saline (PBS, Thermo Fisher Scientific) and placed on wet ice before immediate analysis. Cells were re-suspended in a solution of 50 µg/mL of propidium iodide (PI; Thermo Fisher Scientific) to evaluate mortality of the target cells immediately prior to acquisition using two-color (DiO vs. PI) flow cytometry. The fluorescence of at least 1000 target cells was read using a FACScan flow cytometer (Becton, Dickinson, and Co.) and the automated CellQuest software (Becton Dickinson Immunocytometry System, San Jose, CA, USA). Effector cells were identified by their relative size (forward-scattered light) and their complexity (side-scattered light) and distinguished from DiO-labeled target cells, which show higher fluorescence at 530 nm (FL-1). Dead or dying cells incorporate PI due to membrane instability and show higher fluorescence at 630 nm (FL-3). Results were calculated as:

(2) $$\mathrm{Percent}\;\mathrm{target}\;\mathrm{cell}\;\mathrm{mortality}\;=\;\left(\frac{\#\;dead\;target\;cells}{\#\;dead\;target\;cells\;+\;\#\;live\;target\;cells}\right)\;\times \;100\;$$

The percent of spontaneous target cell mortality was then subtracted from the percent target cell mortality to calculate specific target cell mortality.

### 2.8. Statistical Analyses

Statistical analyses were conducted using SPSS (v27, SPSS Inc., Chicago, IL, USA). One-way analysis of variance (ANOVA) was used to compare turtle body mass, SCL, and BCI by sampling site and tumor score using transformations as necessary to meet test assumptions, followed by Tukey’s post hoc tests with Bonferroni correction. The relationship between log-transformed SCL and body mass was assessed using linear regression. For binary data (e.g., presence/absence of FP, leeches, barnacles, injuries, and presence/absence of ChHV5 DNA or antibodies), chi-square analysis or Fisher’s exact tests (when *N* ≤ 5) were employed using Bonferroni correction for multiple comparisons. Independent samples t-tests were used to compare (1) calculated BCI between two subjective body condition scores (good versus robust) and (2) natural killer cell activity between turtles with and without FP. Cohen’s Kappa (κ) coefficient was calculated to determine the qualitative level of agreement between FP presence/absence and qPCR and ELISA results for ChHV5, and to compare qPCR to ELISA results for ChHV5 [75]. Logistic regression was used to determine if turtle size (i.e., SCL) was related to seroprevalence (i.e., positive and negative). Due to a lack of normality in lymphocyte proliferation assay data, Kruskal–Wallis tests with post hoc Dunn’s tests were used to calculate differences in lymphocyte proliferation by sampling location. Because there were no differences in lymphocyte proliferation by study site for non-tumored turtles and for all turtles combined, all sites were combined for comparisons of lymphocyte proliferation activity with regards to the presence/absence of FP, FP tumor severity, and ChHV5 seroprevalence.

## 3. Results

### 3.1. Physical Examination and Morphometrics

A total of 45 green turtles were captured from three study sites including Big Bend (*N* = 16), Jennings Cove (*N* = 18), and Jupiter Inlet (*N* = 11). All turtles were considered to be immature based on SCL measurements (range: 26.0–70.1 cm) [7,76]. Straight carapace length (*F*(2, 42) = 13.840; *p* < 0.001) and body mass (*F*(2,40) = 13.623; *p* < 0.001) differed by capture site, as turtles captured at Jennings Cove were significantly larger and heavier than turtles captured at Big Bend (*p* < 0.001) and the Jupiter Inlet (*p* < 0.001). Log-transformed body mass and SCL were very strongly correlated (y = 3.07x − 3.99; *r*^2^ = 0.99; *p* < 0.001). Neither SCL nor body mass differed by tumor score category (*p* > 0.05 in both cases). Calculated BCI did not differ by capture site (*p* > 0.05), but BCI was significantly higher (*t*(42) = 2.243; *p* = 0.030) in turtles assigned a subjective body condition score of “robust” (mean ± standard deviation (SD) = 1.34 ± 0.11) compared to those given a score of “good” (mean ± SD = 1.27 ± 0.10). All captured turtles were considered to be of “good” (*N* = 30) or “robust” (*N* = 15) body condition, with no significant differences by site after Bonferroni correction. No sampled animals were score as “thin” or “emaciated” based on subjective observation.

*Ozobranchus* leeches, leech cocoons, and barnacles (*Chelonibia* spp.) were present on the skin and/or carapace of 22% (10/45), 20% (9/45), and 64% (29/45) of turtles, respectively. Leech (*Χ*^2^(2) = 13.705; *p* = 0.001) and leech cocoon (*Χ*^2^(2) = 11.540; *p* = 0.003) prevalence differed by site, wherein Jennings Cove turtles had a higher prevalence of leeches (*p* = 0.001) and leech cocoons (*p* = 0.003) in comparison to turtles from Big Bend. No differences in leech prevalence were observed between turtles from Jupiter Inlet and turtles from Big Bend or Jennings Cove (*p* > 0.05 in both cases).

Flipper damage was observed on 16% (7/45) of turtles, including small and large notches of unidentified cause (*N* = 4), as well as partial (*N* = 2) and complete amputations (*N* = 1) of at least one limb. Carapace damage was observed on 11% (5/45) of turtles and included a small notch of unknown origin to the left posterior carapace (*N* = 1), a possible healed shark predation injury (*N* = 1), and healed caudal vessel strikes (*N* = 3). Prevalence of flipper and carapace damage did not differ by site or between turtles with and without FP (*p* > 0.05 in both cases). A complete description of morphometrics, BCI, epibiota prevalence, and flipper and carapace damage prevalence is reported in Table 1.

### 3.2. Fibropapilloma Tumor Score and Severity

The overall prevalence of FP tumors across all three study sites was 56% (25/45), with the highest prevalence observed in turtles from Big Bend (69%; 11/16), followed by Jennings Cove (56%, 10/18), and then Jupiter Inlet (36%, 4/11); however, these differences were not statistically significant (*p* > 0.05). Balazs–Work tumor scores ranged from 0 to 3 across all sites; median tumor scores were 0 (Jupiter Inlet), 1 (Big Bend), and 2 (Jennings Cove). These results failed to achieve statistical significance, but it is worth noting that only Jennings Cove had turtles in the tumor score 3 category (*N* = 3) and also had no turtles of tumor score 1. Lastly, total tumor number per turtle ranged from 0 to 70; turtles from Jennings Cove had the most tumors (median = 13), followed by Big Bend (median = 3), and then Jupiter Inlet (median = 0). Again, these results did not significantly differ by site (*p* > 0.05).

**Figure 2:**
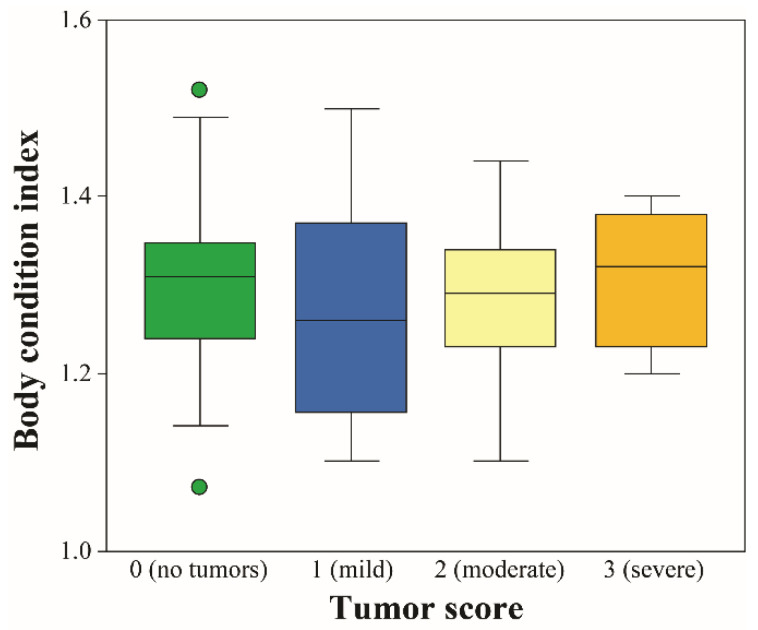
Body condition index by tumor score category in green turtles (*Chelonia mydas*) from Florida, USA. The central boxes represent the lower to upper quartiles, with the middle line representing the median. The vertical lines extend from the minimum to maximum values. Circles indicate “outside” values that are smaller or larger than the lower or upper quartile, minus or plus 1.5-fold the interquartile range, respectively. No significant differences were observed between the different tumor score groups at *p* < 0.05.

Tumor score, severity, and total tumor number did not differ among turtles of different body sizes (*p* > 0.05), likely due to low sample sizes. Turtles in the 20–29.9 cm size range (*N* = 7) had the lowest FP prevalence, severity, and tumor number, which increased in turtles of 30–39.9 cm (*N* = 21) and 40–49.9 cm (*N* = 6), then decreased in turtles in the 50–59.9 cm size range (*N* = 8). Turtles in the >60 cm size range (*N* = 3) showed no external evidence of tumors. A complete description of FP prevalence, severity, and tumor number is reported in Table 1. Neither BCI nor subjective body condition significantly differed between turtles with and without FP (*p* > 0.05 in both cases). More specifically, BCI did not differ by Balazs–Work tumor score category (*N* = 19 for tumor score 0; *N* = 8 for tumor score 1; *N* = 14 for tumor score 2; *N* = 3 for tumor score 3; *p* > 0.05) (Figure 2). Prevalence of leeches, cocoons, and barnacles did not differ between turtles with and without FP (*p* > 0.05).

### 3.3. Molecular Detection of ChHV5 and ChHV6 DNA and Antibodies

Blood samples from 1/40 (3%) and 0/40 (0%) turtles tested positive for ChHV5 and ChHV6 DNA via qPCR, respectively. The amplified DNA sequence from the single sample (a turtle from Big Bend with a tumor score of 2) that tested positive for ChHV5 DNA was subjected to Sanger sequencing, and the resulting trace file matched to the ChHV5 partial genome (GenBank accession number HQ878327.2) with ≥99% identity. This sample had 130 viral copies/μg DNA.

In total, 8/40 (20%) of turtles tested positive for antibodies to ChHV5, with seroprevalence highest in turtles from Jennings Cove (46%, 6/16), followed by Big Bend (13%, 2/15), and Jupiter Inlet (0%, 0/9). These results were not statistically significant after Bonferroni correction. The single turtle that tested positive for ChHV5 DNA by qPCR tested negative for antibodies to the ChHV5 peptide. One turtle from Jennings Cove (3%; 1/37) tested positive for antibodies to ChHV6; this individual had no external tumors, but also tested positive for antibodies to ChHV5. Fibropapilloma status (i.e., tumored or non-tumored) and FP tumor score were not significantly related to ChHV5 antibody prevalence (*p* > 0.05); however, the seroprevalence rate was 43% (6/14) in turtles with tumor scores 2 and 3, compared to 13% (3/21) seroprevalence in turtles with tumor scores 0 and 1. Using logistic regression, we observed a significantly positive association (*p* = 0.034) between ChHV5 seropositivity and turtle size (i.e., SCL). For each 1 cm increase in SCL, turtles were 1.098-fold (95% CI = 1.007–1.197) more likely to test positive for antibodies to ChHV5.

Cohen’s κ coefficient revealed poor agreement between: (1) FP status and ChHV5 qPCR results (κ = 0.041; 95% CI = −0.039–0.121; *p* > 0.05); (2) FP status and ChHV5 ELISA results (κ = 0.070; 95% CI = −0.180–0.320; *p* > 0.05); and (3) ChHV5 PCR and ELISA results (κ = −0.050; 95% CI = −0.141–0.040; *p* > 0.05). Cohen’s κ coefficient was not calculated for ChHV6 qPCR and ELISA results, as all qPCR results were negative for ChHV6.

### 3.4. Relationships of Fibropapillomatosis and ChHV5 Seroprevalence to Lymphocyte Proliferation Assays

**Figure 3:**
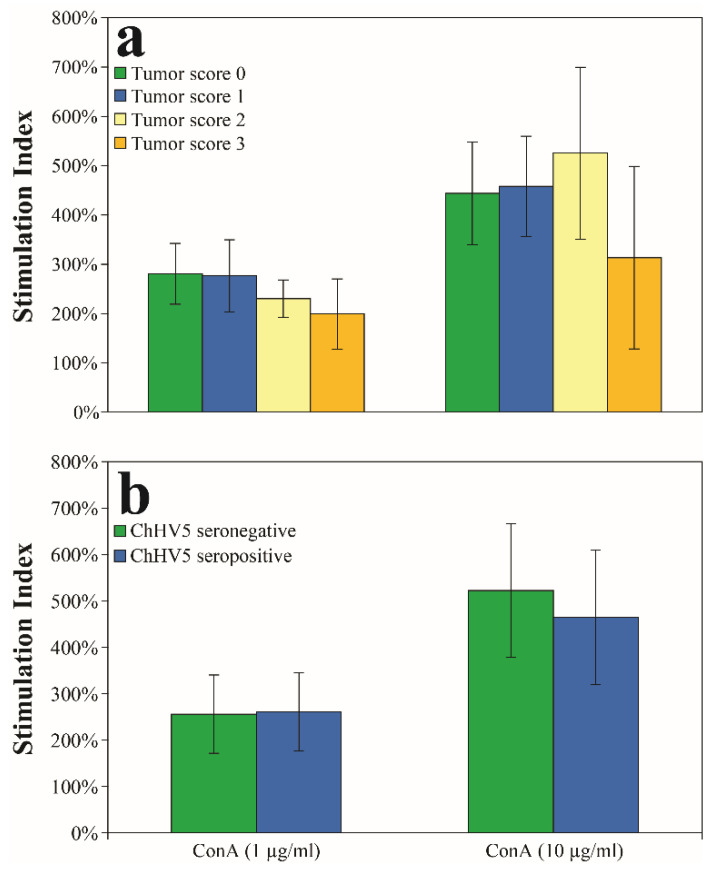
In vitro mitogen-induced lymphocyte proliferation in green turtles (*Chelonia mydas*) from Florida, USA. T-cell mitogen concanavalin A (ConA) results are shown for suboptimal (ConA: 1 μg/mL) and optimal (ConA: 10 μg/mL) stimulation concentrations (mean ± standard error) for comparisons to (**a**) fibropapilloma tumor score and (**b**) ChHV5 serology. No significant differences were observed between the different tumor score groups or between seronegative and seropositive turtles at *p* < 0.05.

Lymphocyte proliferative responses by capture location were compared in two ways: (1) in turtles with no external tumors (i.e., tumor score 0) and (2) in all turtles (i.e., tumor scores 0–3). In non-tumored turtles, no differences between sites were observed for the T-cell mitogen ConA (*p* > 0.05). No differences between sites were observed for ConA when all turtles, regardless of tumor score, were included (*p* > 0.05 in all cases). All sites were therefore combined for statistical analysis of lymphocyte proliferation assays in relation to tumor score (tumor score 0, *N* = 17; tumor score 1, *N* = 8; tumor score 2, *N* = 11; tumor score 3, *N* = 3). Proliferation results for both suboptimal and optimal stimulation concentrations of ConA showed no differences between turtles with or without FP, by tumor score category (Figure 3a), or between ChHV5 seronegative (*N* = 24) and seropositive turtles (*N* = 8; Figure 3b) (*p* > 0.05 in all cases).

### 3.5. Relationship of Fibropapillomatosis to Natural Killer Cell Activity

**Figure 4:**
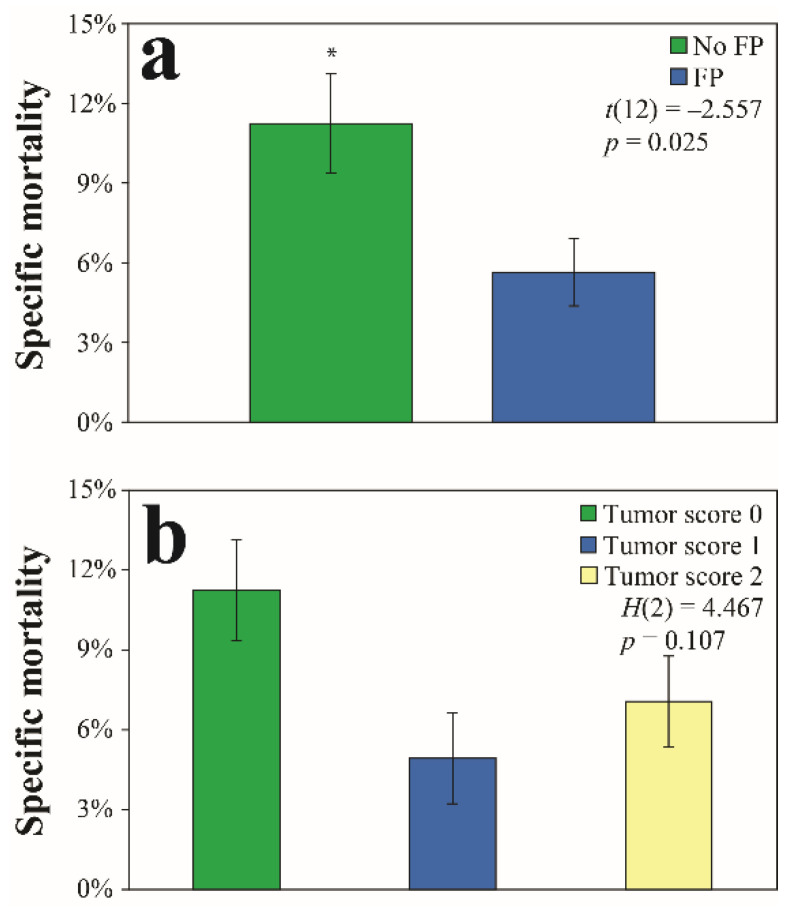
In vitro natural killer cell activity in green turtles (*Chelonia mydas*) with and without fibropapillomatosis (FP) from Florida’s Big Bend, USA measured as a percentage of specific mortality (mean ± standard error). A ratio of effector cells (i.e., natural killer cells) to target cells (i.e., YAC-1 tumor cell lines) of 50:1 was chosen. (**a**) Natural killer cell activity was significantly higher in turtles (at *p* < 0.05) without fibropapilloma (FP) tumors, designated by the asterisk. (**b**) No statistically significant differences in natural killer cell activity by tumor score were observed.

Comparisons of natural killer cell activity were compared in two ways in turtles from Big Bend: (1) between tumored (*N* = 9) and non-tumored (*N* = 5) turtles and (2) by tumor score (*N* = 5 for tumor score 0; *N* = 6 for tumor score 1; *N* = 3 for tumor score 2). Natural killer cell activity was significantly lower in turtles with FP compared to turtles without FP at an effector to target cell ratio of 50:1 (no FP mean ± SD: 11.2 ± 4.2%; FP mean ± SD: 5.6 ± 3.8%; *t*(12) = −2.557; *p* = 0.025) (Figure 4a). No significant differences by tumor score were observed for natural killer cell activity (*H*(2) = 4.467; *p* = 0.107) at an effector to target cell ratio of 50:1 (Figure 4b).

## 4. Discussion

### 4.1. Fibropapillomatosis in Green Turtles from Florida

Fibropapillomatosis is considered a panzootic in green turtles [77] that is likely transmitted horizontally once juvenile turtles recruit back to neritic habitats [19,36,78]. Overall prevalence of FP in green turtles in this study was 56% (25/45) and was highest in turtles from Big Bend (69%; 11/16), followed by Jennings Cove (56%; 10/18), and Jupiter Inlet (36%; 4/11). Although not statistically significant, results of FP prevalence and severity based on 10 cm size-class distributions follow what is previously known for green turtles, wherein FP prevalence, severity, and total tumor number are highest in green turtles ranging from 30 to 59.9 cm SCL [13]. Despite the low sample sizes of turtles captured in each foraging aggregation, our results are similar to prior studies. High prevalence of FP in Florida’s green turtles has been previously reported in Big Bend (66%), within the IRL near Sebastian Inlet (up to 72%) and Jennings Cove (up to 90%), and in Lake Worth Lagoon (up to 79%) [14,16,18,65,79,80]. Despite this high occurrence in certain areas of Florida, several locations in the state host green turtle aggregations with few to no tumors including the Trident Submarine Basin (0–18%), the Key West National Wildlife Refuge (6%), turtles entrained in the St. Lucie Power Plant (up to 2–13%), and nearshore reef sites in central Florida (8–34%) [14,65,79,80,81]. Typically, high prevalence of FP is reported in poorly circulated lagoon systems that occur near heavily urbanized areas with a number of point- and nonpoint-sources of agricultural and urban effluent [13,14,16,18,19,36]. Therefore, it is expected that FP prevalence rates would be high in certain areas of Florida (e.g., IRL and Lake Worth Lagoon); however, Florida’s Big Bend has one of the highest documented FP prevalence in the state, with some studies reporting rates >80% (albeit with low sample sizes) [17,26]. This finding is unexpected as this area has natural land coverage, numerous conservation protections of its coastal and inland areas, a low human population density, and infrequent occurrences of harmful algal blooms that generate potential tumor-promoting biotoxins [17,22,23,26,58,59]. Four rivers empty into the Big Bend study site both locally (Crystal, Homosassa River, and Chassahowitzka Rivers) and regionally (Fenholloway River), and may carry contaminants from anthropogenic sources that act as FP cofactors [18,82]. Green turtles in Puerto Rico have also shown a higher prevalence of FP in more pristine sites in comparison to areas with increased human development [83]; however, reasons for this remain unknown. Turtles from more pristine sites are therefore not necessarily at a decreased risk of developing FP, and other cofactors besides habitat quality may play a role in FP transmission and pathogenesis. Lastly, presence of leeches or cocoons was not significantly related to FP prevalence, which does not support the hypothesis that *Ozobranchus* leeches are vectors of ChHV5 [84,85].

Despite a high prevalence of FP in turtles captured in Big Bend, tumor severity and number were highest in turtles captured in Jennings Cove (although not significantly so, likely due to small sample sizes). These findings are similar to other studies in Florida, whereby tumor severity was highest in the IRL compared to a nearshore Sabellariid worm reef and the Trident Submarine Basin. More severe FP in the IRL may be explained by size, environmental, dietary, or genetic differences between individuals in these areas [86]. Another potential explanation related to dissimilarities in FP severity may be due to ChHV5 viral variants that are present in these areas, as multiple studies have shown that different variants can lead to different presentations/severity of disease [35,36,78,87,88,89,90]; however, viral variants were not assessed in this study.

Body condition index of green turtles did not differ by tumor score category, and all animals sampled for this study were given subjective body condition scores of “good” or “robust.” In green turtles from Hawaii, Brazil, and Taiwan, no differences in BCI were observed between FP tumor score categories [24,49,91,92,93]. Interestingly, BCI in Brazilian green turtles was significantly higher in those with FP (all tumor scores combined) compared to FP-free turtles. This was attributed to tumors’ mass contributing to increased body mass, and therefore higher BCI [92]; however, a more likely explanation for our results is that adequate forage items are available in the locations examined here, allowing for the maintenance of good body condition [24]. This finding is corroborated by observations of food present during esophageal lavage (conducted for a separate study) in all turtles sampled during this study. It is also possible that Fulton’s BCI is not an adequate method for detecting differences in turtles with and without FP [47,91] and that subjective estimates (e.g., emaciated, thin, good, and robust) may be better for assessing body condition declines with increasing tumor score [49]. It is noteworthy that only three turtles of tumor score 3 were captured during this study, which prevented rigorous comparisons of BCI to the other tumor score groups.

### 4.2. Diagnostic Assays for ChHV5 and ChHV6

Detection of ChHV5 DNA in blood (i.e., DNAemia) indicates the presence of circulating ChHV5 DNA, suggesting active or recrudescent infection [40]. Despite an overall FP prevalence of 56% in green turtles captured during this study, only one individual from Florida’s Big Bend tested positive for ChHV5 DNA in whole blood using qPCR. Low prevalence of ChHV5 in blood samples of turtles with FP is not uncommon as has been observed in previous studies of wild green turtles from the IRL (17%; 33/196) [93] and rehabilitating green turtles at three sea turtle hospitals across the southeastern United States (33%; 7/21) [40]. Low ChHV5 DNA prevalence values were also reported in sea turtles without external evidence of FP, including wild (0%, 0/20), and rehabilitating (14%, 7/52) green turtles from North Carolina, nesting green turtles from Juno Beach, Florida (8%, 5/60), and wild-caught green turtles from Grenada (0%, 0/55) [4,44,94]. Therefore, we expected only a few turtles to test positive for ChHV5 via qPCR, as many herpesviruses become latent within healthy hosts and therefore do not necessarily show relationships with herpesviral DNAemia [4,40]. It is also possible that FP tumors represent niduses for the virus, resulting in low circulating numbers of ChHV5 in the blood [40].

An additional and likely more sensitive method to determine past infection with ChHV5 is through serology, as this method allows for the detection of circulating antibodies to ChHV5 [95]. Once turtles recruit back to neritic habitats, they are more likely to be exposed to ChHV5 [78]. Previous studies of juvenile and subadult green turtles with and without FP captured in three locations across Florida’s east coast demonstrated a seroprevalence for antibodies to ChHV5 of 84% (143/171) [72]. In the present study, seroprevalence was 20% (8/40) for all green turtles, which did not significantly differ by capture location; however, green turtles captured at Jennings Cove had the highest seroprevalence rate (46%; 6/16). This supports our findings of both higher tumor numbers and increased seroprevalence with turtle size, as turtles from Jennings Cove were significantly larger than turtles from Big Bend and Jupiter Inlet (Table 1). Green turtles with experimentally transmitted FP developed antibodies to ChHV5 one year after inoculation; thus, turtles exposed to ChHV5 in near-shore habitats may not immediately test positive for antibodies to ChHV5 [21]. Seronegativity in turtles with external FP tumors could occur if (1) the tumor is antigenically restricted, (2) the virus is “trapped” in but not replicating in tumors (i.e., no immunologic stimulation), (3) immunosuppression is preventing a detectable antibody response, and/or (4) FP-positive, serologically negative turtles are infected with a different serotype of ChHV5 that is not detected with the ELISA used in this study [96,97]. A lower seroprevalence rate of 29% (12/41) was observed in nesting green turtles from Juno Beach, Florida, suggesting that antibody titers may decrease over time post-infection as turtles grow and age, and the virus goes into latency [4]. Virus–tumor interactions may also vary geographically, as green turtles from Florida will often show seropositivity independent of tumor status, whereas most seropositive turtles from Hawaii have tumors. This was attributed to dissimilar behaviors between the two populations, which may have led to the formation of different viral strains with differing pathogenicities [98].

Prevalence of antibodies to ChHV5 was higher in turtles with tumor scores 2 and 3 (43%) compared to 13% in turtles with tumor scores 0 and 1, although this finding was not significant. Increased sample sizes would have likely revealed a significant trend, since turtles in more advanced stages of FP have had adequate time to develop antibodies to ChHV5 [21,55,98]. We also observed low diagnostic agreement between turtles that tested positive for ChHV5 DNA using qPCR and those that tested positive for antibodies to ChHV5, similar to that previously shown in nesting green turtles from Juno Beach, Florida, indicating that these individuals were in different stages of infection (i.e., viral DNAemia as determined by PCR and previous infection as determined by ELISA) [4]. Therefore, we recommend that PCR and serology be utilized simultaneously to best understand ChHV5 infection and exposure [4].

No turtles in this study tested positive for ChHV6 DNA, and just one individual from Jennings Cove tested positive for antibodies to ChHV6. This individual was larger than average compared to other turtles captured for this study (SCL = 49.0 cm), had no external evidence of FP, and also tested positive for antibodies to ChHV5, indicating exposure to both viruses at some point in the turtle’s life [4,54,72]. Lung-eye-trachea disease associated with ChHV6 infection has never been identified in free-ranging green turtles since it was first identified in maricultured green turtles from the Cayman Turtle Farm in 1975 [53]. Individuals can be exposed and develop antibodies to viruses independently of clinical disease, however, and previous studies have shown that wild green and loggerhead turtles from Florida carry antibodies to ChHV6 at prevalence rates of 10–22% [4,55,95]. No differences in population-level ChHV6 seroprevalence rates were observed over a three-year period in green turtles from Florida, suggesting that ChHV6 exposure was not increasing over time [55]. Our results corroborate these findings. Viral seroconversion and seroreversion within individual turtles have also been observed over relatively short time periods of 5–42 days [44,55]; therefore, some turtles in this study that were previously exposed to ChHV6 (and also ChHV5) may have gone undetected. The suggested transmission mode of ChHV6 is horizontally through seawater, as the virus has been shown to remain infectious for up to five days in this medium with an inverse relationship with water temperature. Horizontal viral transmission via direct contact between turtles and with sediments is also probable [95,99]. Further studies on seroprevalence of ChHV5 and ChHV6 in other sea turtle species from Florida are warranted, as 100% (7/7) of loggerheads in Big Bend tested positive for antibodies to both viruses [100] and foraging grounds serve as areas that facilitate viral transmission [20].

### 4.3. Lymphocyte Proliferation

Lymphocyte proliferation assays have been utilized in previous studies in an effort to better understand immune function in relation to FP prevalence and/or severity. These studies found reduced T-cell (i.e., cell-mediated immunity) and B-cell (i.e., humoral immunity) proliferative responses in turtles with FP [46], with significant declines in moderately (i.e., tumor score 2) and severely afflicted (i.e., tumor score 3) turtles [47]. Additional changes in hematologic and biochemical parameters include anemia, increased corticosterone, higher heterophil:lymphocyte ratios, heterophilia, hypoproteinemia, and bacteremia, suggesting chronic antigenic stimulation, immunosuppression, and/or chronic stress in relation to FP [47,48,101,102]. No differences were observed, however, between turtles with and without FP in regards to phagocytosis and oxidative burst, which are indicators of innate immune function and antimicrobial defense, respectively [103]. Here, we found no differences in T-cell proliferation between turtles with or without FP, by tumor score (Figure 3a), or between ChHV5 seropositive and seronegative turtles (Figure 3b); yet, severely-afflicted turtles (*N* = 3) did show reduced T-cell proliferation by 159%, on average, in comparison to the other three groups. Larger sample sizes of turtles in this group could have likely revealed a significant trend. In a recent study, high concentrations of CD3+ lymphocytes and upregulation of leukocyte and lymphatic processes were observed in early-stage tumors, indicating a measurable immune response during earlier stages of tumor growth. Conversely, in turtles that did not survive rehabilitation with late-stage tumors, CD3+ lymphocytes were less concentrated with an observed downregulation of immune- and apoptotic-related genes [104]. Taken together, the evidence suggests that turtles with FP mount an immune response to infection at least until the moderate stages of the disease, followed by a decrease in immune function. Because moderately-afflicted turtles (i.e., tumor score 2) in this study showed the highest proliferative responses for the optimal concentration of the T-cell mitogen (ConA) and all were in good or robust body condition, it is likely that immunosuppression as a result of advanced stages of the disease occurs after late-stage disease as tumor burdens become severe [47]. This argument is especially convincing as immunosuppression is not a prerequisite for the formation of viral-related diseases [20,95] and sufficient and appropriate nutritional input diminishes the immunosuppressive effects of FP [105,106]. Lastly, these results can lend insight into treatment options for turtles with FP/ChHV5 infection through preventative or therapeutic cancer vaccines using tumor antigen specific T-cells [27].

### 4.4. Natural Killer Cell Activity

Natural killer cells are innate, cytotoxic lymphocytes that function to recognize and clear tumor and virus-infected cells, including a well-characterized response against herpesviruses [73,107,108,109]. Their protective mechanisms lie within the production of cytokines that work towards eliminating pathogens and generating antigen-specific immune responses [110]. Many herpesviruses have developed mechanisms to evade natural killer cell elimination and are known to suppress natural killer cell activity [111]. Only a handful of studies to date have examined natural killer cell activities in turtles, which suggest differences by sex and season, and that activity can decrease upon exposure to certain contaminants [74,112,113]; however, no studies have examined natural killer cell activity in response to disease in sea turtles. Interestingly, several natural killer cell-related cytotoxicity genes were found to be upregulated in FP tumors from juvenile green turtles undergoing rehabilitative care [114]. Additionally, several putative proteins observed in FP tumors that are considered atypical of alphaherpesviruses (e.g., F-lec1 and F-lec2) are associated with activation or inhibition of natural killer cells [115]. Lastly, lymphocytic inflammatory infiltrate, including CD3+ T-cells, is associated with FP tumor tissues [104]. These findings suggests that active inflammatory processes occur within and around FP tumors in response to the high viral load found in tumor tissues compared to healthy, non-tumored tissue [40]. In this study, natural killer cell activity was significantly lower in green turtles with FP compared to turtles without FP (Figure 4a). Collectively, these results indicate that FP tumor development is associated with first an increase, then a subsequent exhaustion of innate immune responses including natural killer cell activity. This depletion of innate immunity may lead to susceptible and/or infected cells becoming more permissive to ChHV5 infection and neoplasia development, respectively. More specifically, ChHV5 infection and FP development may lead to suppression of natural killer cell activity, as has been described with human cancers and infectious and autoimmune diseases. Additionally, depletion of natural killer cells led to enhanced tumor formation in *in vivo* mouse tumor models [116]. More research is needed to better understand how natural killer cells and other immune responses influence FP disease pathogenesis and prognosis.

## 5. Conclusions

Although there is no clear trend in the occurrence or severity of fibropapillomatosis in Florida, and there is no evidence that the disease is a major cause of mortality for afflicted animals in this area, >20% of green turtles stranding in southern Florida have FP [13,14,98]. Therefore, advancing our understanding of FP and its impacts on sea turtle health and survival remains an important research priority for sea turtle conservation [36,117]. Lymphocyte proliferative responses of green turtles from this study suggest that turtles in Florida are able to mount sufficient immune responses after the development of tumors, at least until the disease becomes severe. These results support the hypothesis of Work et al. [47] that immunosuppression occurs after disease onset. We propose that adequate forage availability and good-to-robust nutritional status may help prevent severe immunosuppression, at least until the moderate stages of the disease, as the results of our lymphocyte proliferation assays differed from previous studies showing immunosuppression in the more advanced stages of disease [46,47]. Lastly, we observed that natural killer cell activity was significantly reduced in turtles with FP in comparison to tumor-free turtles, lending further insight on immune system function in relation to FP. It is likely that FP tumor development involves factors related to host and viral biology and physiology, in addition to environmental cofactors [20,21,48].

## Figures and Tables

**Table 1:** Morphometrics and results of external physical exam for green turtles (*Chelonia mydas*) from three locations in Florida, USA. For standard straight carapace length (SCL), body mass, body condition index (BCI), tumor score, and tumor number, mean ± standard deviation are given with the median and range in parentheses. Sample sizes differed in some categories as not all analyses were conducted on every sample; this is indicated parenthetically where relevant. Different superscript letters next to each category represent significant differences between sites, when present. Abbreviations: ChHV5, chelonid alphaherpesvirus 5; ChHV6, chelonid alphaherpesvirus 6; qPCR, quantitative polymerase chain reaction.

Physical Examination	Big Bend	Jennings Cove	Jupiter Inlet	Total
*N*	16	18	11	45
Sampling dates	Aug 2020	Sep 2018–Oct 2020	Sep 2020	Sep 2019–Oct 2020
SCL (cm)	34.4 ± 4.6 ^A^ (33.8) (27.9–43.4)	48.2 ± 10.3 ^B^ (49.8) (32.5–70.6)	35.4 ± 9.1 ^A^ (32.1) (27.2–53.0)	40.2 ± 10.6 (36.9) (27.2–70.6)
Body mass (kg)	5.6 ± 2.7 ^A^ (5.2) (2.8–12.3)	16.8 ± 11.6 ^B^ (14.4) (4.5–46.8)	6.7 ± 6.4 ^A^ (3.8) (2.8–19.5)	10.2 ± 9.4 (6.6) (2.8–46.8)
Body condition index	1.29 ± 0.11 (1.31) (1.10–1.50)	1.32 ± 0.12 (1.31) (1.07–1.52)	1.26 ± 0.09 (1.27) (1.10–1.39)	1.29 ± 0.11 (1.31) (1.07–1.52)
Subjective body condition	Good: 44% (7/16) Robust: 56% (9/16)	Good: 72% (13/18) Robust: 28% (5/18)	Good: 91% (10/11) Robust: 9% (1/11)	Good: 67% (30/45) Robust: 33% (15/45)
Leech prevalence	0% (0/16) ^A^	50% (9/18) ^B^	9% (1/11) ^AB^	22% (10/45)
Leech cocoon prevalence	0% (0/16) ^A^	44% (8/18) ^B^	9% (1/11) ^AB^	20% (9/45)
Barnacle prevalence	63% (10/16)	61% (11/18)	73% (8/11)	64% (29/45)
Flipper damage	6% (1/16)	22% (4/18)	18% (2/11)	16% (7/45)
Carapace damage	19% (3/16)	11% (2/18)	0% (0/11)	11% (5/45)
**FP and diagnostic assays**	**Big Bend**	**Jennings Cove**	**Jupiter Inlet**	**Total**
FP prevalence	69% (11/16)	56% (10/18)	36% (4/11)	56% (25/45)
Balazs–Work tumor score	1.0 ± 0.8 (1) (0–2)	1.3 ± 1.2 (2) (0–3)	0.5 ± 0.8 (0) (0–2)	1.0 ± 1.0 (1) (0–3)
Tumor number	10 ± 15 (3) (0–44)	21 ± 25 (13) (0–79)	4 ± 8 (0) (0–26)	13 ± 20 (2) (0–79)
ChHV5 qPCR+	6% (1/16)	0% (0/13)	0% (0/11)	3% (1/40)
ChHV5 seropositive	13% (2/15)	46% (6/16)	0% (0/9)	20% (8/40)
ChHV6 qPCR+	0% (0/16)	0% (0/13)	0% (0/11)	0% (0/40)
ChHV6 seropositive	0% (0/15)	8% (1/13)	0% (0/9)	3% (1/37)

## Data Availability

The data sets utilized for this study can be made available by the corresponding author upon reasonable request.
